# High-density lipoprotein-related inflammatory markers and their association with all-cause and cardiovascular mortality in an ageing population: findings from a prospective cohort study based on NHANES data

**DOI:** 10.7189/jogh.16.04107

**Published:** 2026-03-27

**Authors:** Tongtong Hu, Runyang Chen, Yang Gu, Chunyang Yu, Xingjing Liu, Xiwen Zhang

**Affiliations:** 1 Department of Cardiology, the Affiliated Huaian No. 1 People’s Hospital of Nanjing Medical University, Huai’an, Jiangsu Province, China; 2 Department of Endocrinology, the Affiliated Huaian No. 1 People’s Hospital of Nanjing Medical University, Huai’an, Jiangsu Province, China

## Abstract

**Background:**

High-density lipoprotein cholesterol (HDL-C)-related inflammatory markers are increasingly being recognised as indicators of inflammation and metabolism associated with cardiovascular events. Here, we examined their associations with all-cause and cardiovascular disease (CVD) mortality in the ageing population.

**Methods:**

We retrieved data on patients aged ≥60 years from the National Health and Nutrition Examination Survey (2001–2018). We ascertained exposures (neutrophil/HDL ratio (NHR), lymphocyte/HDL ratio (LHR), monocyte/HDL ratio (MHR), platelet/HDL ratio (PHR)) and covariates at baseline and cross-linked them to mortality outcomes *via* the National Death Index. We tested for associations using survey-weighted Cox proportional hazards models, with restricted cubic splines assessing nonlinearity and C-statistics evaluating discrimination.

**Results:**

We included 5700 patients in our sample. A total of 1817 deaths occurred over a mean follow-up of 7.51 years, including 618 CVD deaths. After multivariable adjustment, higher NHR showed a consistent linear association with increased all-cause and CVD mortality. Each standard deviation increase in NHR corresponded to 11% higher all-cause mortality (hazard ratio (HR) = 1.11) and 12% higher CVD mortality (HR = 1.12). Compared to the lowest tertile, the highest NHR tertile was associated with 29% higher all-cause mortality (HR = 1.29) and 70% higher CVD mortality (HR = 1.70). Higher LHR showed a non-linear relationship with 21% lower all-cause mortality (HR = 0.79) and 31% lower CVD mortality (HR = 0.69) in the highest tertile. MHR and PHR showed no significant associations with mortality.

**Conclusions:**

Higher NHR was consistently associated with increased all-cause and CVD mortality among older adults, while higher LHR showed an inverse association. NHR may serve as a useful inflammatory-lipid marker for mortality risk assessment in ageing populations.

Cardiovascular diseases (CVD), such as coronary artery disease (CAD), arrhythmia, or congestive heart failure, are characterised by structural or functional abnormalities in the cardiovascular system that disrupt its functioning. They have contributed significantly to the global disease burden during the last several decades, with 523 million cases reported in 2019 [1]. The number of CVD-related deaths has also risen consistently, reaching 18.6 million in 2019 [2]. The majority of CVD patients are elderly individuals aged ≥60 years [2].

Inflammation and lipid metabolism disorders are key factors in the development of CVD. Inflammatory factors, for example, drive the progression of CVD by affecting vascular endothelial function, thereby promoting atherosclerosis and cardiac fibrosis [3–5]. Excessive lipid accumulation, meanwhile, can induce various pathological processes, including mitochondrial damage, reactive oxygen species production, inflammation, and apoptosis, while increased myocardial lipids may have detrimental effects on left ventricular mass and diastolic function [6–8]. Inflammation and lipid metabolism can then interact to exacerbate CVD progression [9].

Blood biomarkers are commonly used to explore the pathogenesis of CVDs. High-density lipoprotein cholesterol (HDL-C), for example, was identified as an anti-arteriosclerosis and vascular protection biomarker [10]; HDL-related inflammatory markers obtained from peripheral blood cell tests and HDL level, including lymphocyte/HDL ratio (LHR), neutrophil/HDL ratio (NHR), monocyte/HDL ratio (MHR) and platelet/HDL ratio (PHR), have been utilised to assess the inflammation and lipid metabolism in several diseases like depression [9], diabetes [11] and chronic kidney disease recently [12]. However, few previous studies have explored the relationship between HDL-related inflammatory markers with CVD in the ageing population.

We aimed to examine the associations of these HDL-C-related inflammatory markers with all-cause and CVD mortality. Based on biological plausibility and prior evidence linking neutrophil-driven inflammation and HDL function to cardiovascular pathogenesis [13.41], we pre-specified the NHR as our primary exposure of interest, and assessed LHR, MHR and PHR as secondary exploratory markers for comparative purposes. We utilised data from the National Health and Nutrition Examination Survey (NHANES) to address these questions in a representative ageing population [14].

## METHODS

Our study aligns with the Journal of Global Health’s GRABDROP guidelines (Table S1 in the **Online Supplementary Document**).

### Study design and populations

We conducted a retrospective analysis of data from the NHANES, a prospective cohort study that conducts repeated cross-sectional surveys designed to assess the health and nutritional status of the non-institutionalised US population [15]. Specifically, we focused on data from the 2001–2018 NHANES cycles. All exposures (inflammatory ratios), covariates, and laboratory measurements were ascertained at the baseline examination for each participant within their respective cycle. We then prospectively followed-up participants for mortality outcomes until their death or 31 December 2019, whichever came first, via linkage of NHANES data with the National Death Index (NDI) [16]. Inclusion criteria were age ≥60 years and complete data on all exposures (lymphocytes, monocytes, neutrophils, platelets, HDL), covariates, sample weights, and mortality follow-up. A detailed flow diagram of participant selection is provided in the Results section and Figure S1 in the **Online Supplementary Document**.

### Assessment of high-density lipoprotein-related inflammatory markers

We selected NHR as the primary exposure variable of interest, and MHR, LHR, PHR as secondary exploratory exposures. We calculated the ratios of HDL-related inflammatory markers directly from laboratory measurements based on cell counts (×10^9^/L) from complete blood counts divided by HDL-C concentration (mmol/L). The NHANES employs standardised procedures with quality control to ensure comparability across survey cycles. We analysed these ratios, which were right-skewed, as continuous variables on their original scale. For the primary analysis, we standardised each ratio to a z-score (mean = 0, standard deviation (SD) = 1); each hazard ratio (HR) corresponds to a one SD increase in the observed distribution. To assess robustness and nonlinearity, we also analysed the ratios as tertiles and by using restricted cubic splines (RCSs) [11].

### Outcome definitions

We defined CVD mortality based on the underlying cause of death recorded in the NDI linkage files, coded with ICD-10 codes I00–I99 (diseases of the circulatory system). This ensured the inclusion of deaths due to heart disease (*e.g.* ischaemic heart disease, heart failure) as well as cerebrovascular disease (stroke, I60–I69) [17].

### Evaluation of covariates

The NHANES gathered data on age, sex, race (Mexican American, White, Black, or other race), education (high school/equivalent, under or above high school), income to poverty ratios (<1, 1, 3, >3), marital status (married or living with a partner, other), smoking status (never, former, current), drinking status (heavy: ≥3 drinks/day for men, ≥2 for women; low-to-moderate: 1–2 drinks/day for men, 1 for women; non-drinker: 0 drinks, based on self-reporting to the NHANES Alcohol Use Questionnaire), body mass index (BMI) (normal, overweight, obesity). Laboratory analyses included total cholesterol (TC), triglyceride (TG), low-density lipoprotein cholesterol (LDL-C) (derived using the Friedewald equation for participants with trygliceride ≤400 mg/dL as per NHANES protocols), fasting glucose, glycated haemoglobin (HbA1c), serum creatinine, and estimated-glomerular filtration rate (eGFR) calculated using the Chronic Kidney Disease Epidemiology Collaboration. Hypertension (yes/no), diabetes (yes/no), and CVD were also assessed. Prevalent CVD was defined as a self-reported, physician-diagnosed composite of coronary heart disease, myocardial infarction, stroke, angina, or heart failure, based on responses to the standard NHANES Medical Conditions Questionnaire [18]. Hypertension was identified when any of the following criteria was met: a systolic blood pressure ≥140 mm Hg or a diastolic blood pressure ≥90 mm Hg; or a self-reported, physician-ascertained diagnosis of hypertension. Diabetes was identified when any of the following criteria were present: a HbA1c ≥6.5%, a fasting blood glucose concentration ≥126 mg/dL, a self-reported, physician-ascertained diagnosis diagnosis of diabetes [19]. Participants diagnosed with CAD, congestive heart failure, arrhythmia, or stroke were categorised with CVD by self-reported physician using a standardised medical condition questionnaire [20].

### Data analysis

All analyses accounted for the complex survey design of NHANES and were conducted using fasting subsample weights, as the laboratory measurements of interest (including HDL-C) were obtained from fasting participants. To create appropriate weights for the pooled analysis across nine survey cycles (2001–2018), we divided the provided two-year fasting subsample weights by the number of cycles (nine), in accordance with NHANES analytic guidelines [21]. We incorporated the survey design variables (specifically, the sampling weights, stratification (strata), and primary sampling units) into all analyses, including descriptive statistics, survival curves, and Cox proportional hazards regression models.

We displayed continuous variables as medians and interquartile ranges, and compared them using a weighted Wilcoxon rank-sum test. We presented categorical variables were presented as frequencies and percentages, and compared them using weighted χ^2^ tests. We categorised participants into tertiles (T1–T3) based on the distribution of each ratio within the overall pooled analytic sample [22]. We employed confounder-adjusted multivariate Cox regression models to evaluate the links of the HDL-related inflammatory markers and mortality, calculating HRs and 95% confidence intervals (CIs) across three models. Model 1 was not adjusted for any covariates. Model 2 was adjusted for age, gender, race, and BMI. Model 3 was adjusted for age, gender, race, education level, family income poverty ratio, smoking status, drinking status, BMI, TC, TG, HbA1c, eGFR, hypertension, diabetes, and CVD.

We generated Kaplan-Meier curves to visualise the cumulative mortality rates across groups and assessed differences using the log-rank test. We also performed subgroup analyses, with sex pre-specified as the primary interaction of interest, and all other interactions (race, BMI, *etc*.) explored descriptively. We assessed nonlinearity using RCSs with three knots (at the 10th, 50th, and 90th percentiles) within survey-weighted Cox models. The HRs from RCSs are expressed relative to the point of minimum estimated risk. We used the C-statistic and its 95% CI as a descriptive measure of discrimination in survey-weighted Cox models. Exploratory reclassification performance was evaluated using bootstrap-based net reclassification improvement [23]. We assessed the proportional hazards assumption for the Cox models using Schoenfeld residuals, tested globally and for the primary exposures (NHR, LHR).

To maintain the representativeness of the target ageing population, which often carries a high burden of pre-existing conditions, our primary analyses included participants with prevalent CVD. We did not conduct additional sensitivity analyses (*e.g.* excluding early deaths or those with baseline CVD) to avoid selection bias and preserve generalisability.

We performed all analyses in R, version 4.2.1 (R Foundation for Statistical Computing, Vienna, Austria). A *P*-value <0.05 was considered statistically significant.

## RESULTS

### Baseline characteristics of the study cohort

We included 91 351 participants from the NHANES data collected between 2001 and 2018. After removing individuals aged <60 years (n = 74 098), those missing data on lymphocytes, monocytes, neutrophils, platelets, or HDL (n = 2126), follow-up (n = 22), covariates or sample weights (n = 9405), we retained 5700 (2853 males and 2847 females) individuals in our analysis (Figure S1 in the **Online Supplementary Document**). Over a mean follow-up of 7.51 years, 1817 (32%) all-cause deaths and 618 CVD deaths occurred (Table S2 in the **Online Supplementary Document**). These individuals had higher TG, Serum creatinine, MHR, NHR, and PHR compared to the survivors. Compared with those in the lowest NHR tertile, individuals with a higher NHR tended to be men and of white ethnicity, and a lower educational attainment, a higher family poverty income ratio, a higher BMI, more current smoker, and a higher prevalence of hypertension, diabetes and CVD (Table S3–6 in the **Online Supplementary Document**).

### Association of LHR, MHR, NHR, and PHR with all-cause mortality in the ageing population

**Figure 1:**
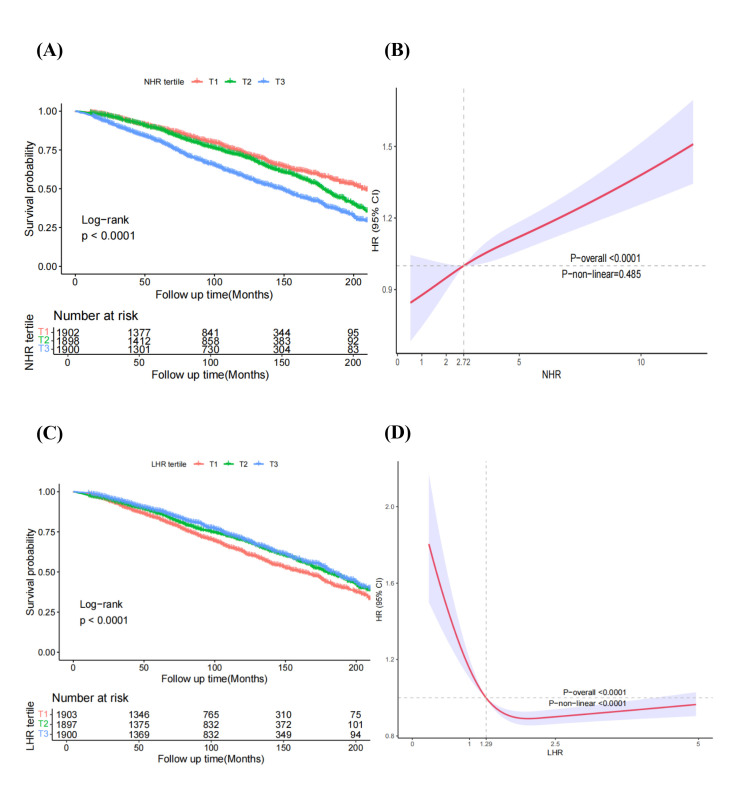
Kaplan-Meier analysis (**Panel A**) and restricted cubic spline (**Panel B**) analysis of all-cause mortality based on NHR groups in the ageing population, and Kaplan-Meier analysis (**Panel C**) and restricted cubic spline (**Panel D**) analysis of all-cause mortality based on LHR groups in the ageing population.

After adjusting for potential covariates in model 3, we found NHR to be associated with an elevated risk of all-cause mortality (*P*-value for trend = 0.006). Using the lowest tertile (T1) as the reference, the HRs for all-cause mortality in the T2 and T3 groups were 0.98 (95% CI = 0.84–1.16) and 1.29 (95% CI = 1.07–1.55), respectively (**Table 1**). Assessment of the proportional hazards assumption using Schoenfeld residuals indicated no significant violation for the primary exposures (NHR: *P* = 0.191; LHR: *P* = 0.557). A minor global deviation (*P* = 0.020) was driven by adjustment covariates (*e.g.* sex, age) rather than the exposures of interest. Additionally, each one-standard-deviation increase in NHR was associated with an increased risk of all-cause mortality in the ageing population after adjusting for confounders (HR = 1.11; 95% CI = 1.09–1.14). Kaplan-Meier survival curves also demonstrated that increased NHR was associated with higher all-cause mortality (log-rank *P* < 0.001) (**Figure 1**, Panel A), while the RCS results showed a linear correlation (*P*-value for nonlinearity >0.05) between the NHR and all-cause mortality in the ageing population (**Figure 1**, Panel B).

**Table 1:** Associations between LHR, MHR, NHR, PHR, and all-cause mortality in the ageing population*

	Model 1		Model 2		Model 3	
	**HR (95% CI)**	***P*-value**	**HR (95% CI)**	***P*-value**	**HR (95% CI)**	***P*-value**
**LHR tertiles**						
*T1*	ref		ref		ref	
*T2*	0.84 (0.74–0.96)	0.012	0.93 (0.82–1.07)	0.311	0.81 (0.70–0.94)	0.004
*T3*	0.86 (0.75–0.98)	0.026	1.03 (0.90–1.18)	0.684	0.79 (0.67–0.93)	0.005
*P–value for trend*		0.022		0.758		0.004
**MHR tertiles**						
*T1*	ref		ref		ref	
*T2*	1.27 (1.10–1.46)	0.001	1.23 (1.06–1.42)	0.006	1.08 (0.93–1.25)	0.319
*T3*	1.73 (1.50–2.00)	<0.001	1.46 (1.26–1.69)	<0.001	1.12 (0.95–1.32)	0.162
*P–value for trend*		<0.001		<0.001		0.171
**NHR tertiles**						
*T1*	ref		ref		ref	
*T2*	1.23 (1.07–1.43)	0.004	1.16 (1.00–1.34)	0.052	0.98 (0.84–1.16)	0.844
*T3*	1.90 (1.65–2.19)	<0.001	1.70 (1.46–1.98)	<0.001	1.29 (1.07–1.55)	0.009
*P–value for trend*		<0.001		<0.001		0.006
**PHR tertiles**						
*T1*	ref		ref		ref	
*T2*	0.84 (0.74–0.96)	0.013	0.94 (0.83–1.06)	0.298	0.86 (0.75 0.98)	0.020
*T3*	0.94 (0.82–1.07)	0.334	1.09 (0.96–1.24)	0.194	0.92 (0.80–1.06)	0.245
*P–value for trend*		0.383		0.186		0.267

CI – confidence interval, HR – hazard ratio, LHR – lymphocyte/high density lipoprotein ratio, MHR – monocyte/high density lipoprotein ratio, NHR – neutrophil/high density lipoprotein ratio, PHR – platelet/high density lipoprotein ratio

*Model 1: unadjusted. Model 2: adjusted for age, gender, race, and BMI. Model 3: adjusted for age, gender, race, education level, family income-poverty ratio, smoking status, drinking status, BMI, TC TG, HbA1c, eGFR, hypertension, diabetes, and CVDs.

Regarding LHR, the Kaplan-Meier survival curves (**Figure 1**, Panel C) showed that increased LHR was associated with decreased all-cause mortality (log-rank *P* < 0.001). Higher LHR was associated with decreased all-cause mortality in models 1 and 3, but not in model 2. For all-cause mortality, using the lowest tertile (T1) as the reference, the HRs in the T2 and T3 groups were 0.81 (95% CI = 0.70–0.94) and 0.79 (95% CI = 0.67–0.93), respectively (P-value for trend = 0.004). The LHR showed a significant nonlinear relationship (*P*-value for nonlinearity <0.001) with mortality (**Figure 1**, Panel D). This explains the significant tertile-based associations, with the inverse link more pronounced at lower LHR levels.

We observed a significant relation between PHR and all-cause mortality only in the T2 group in model 3, but not in the T3 group (*P*-value for trend >0.05). The MHR were not related to all-cause mortality in the ageing population.

### Association of LHR, MHR, NHR, and PHR with CVD mortality in the ageing population

**Figure 2:**
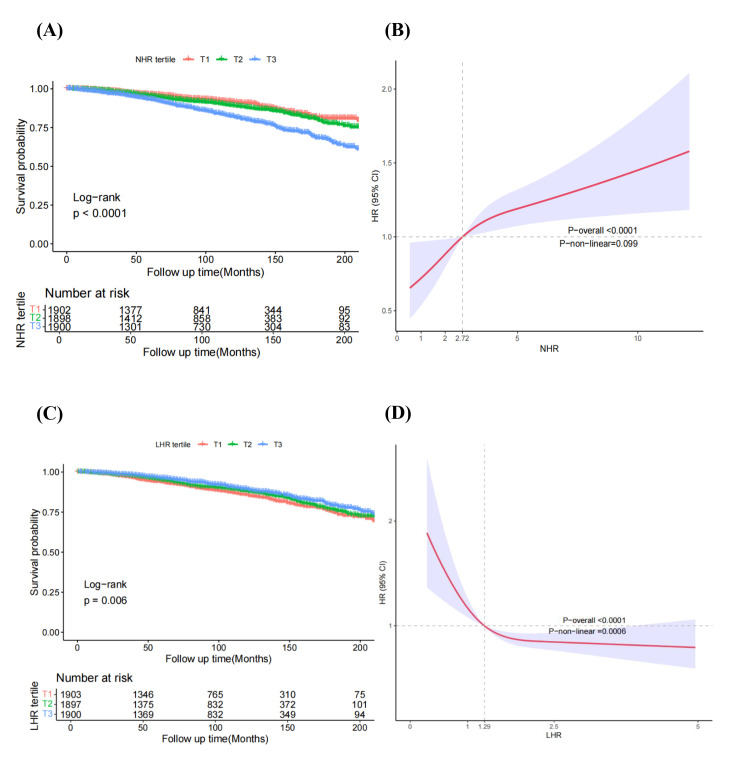
Kaplan-Meier analysis (**Panel A**) and restricted cubic spline (**Panel B**) analysis of CVD mortality based on NHR groups in the ageing population and Kaplan-Meier analysis (**Panel C**) and restricted cubic spline (**Panel D**) analysis of cardiovascular mortality based on LHR groups in the ageing population.

After weighted Cox regression analysis, Kaplan-Meier survival curves displayed that increased NHR was linked to elevated CVD mortality (log-rank *P* < 0.001) (**Figure 2**, Panel A). Using the lowest tertile (T1) as the reference, the multivariate Cox regression analysis indicated that the HRs of NHR were 1.10 (95% CI = 0.82–1.47) and 1.70 (95% CI = 1.23–2.36) for T2 and T3 groups in adjusted model 3, respectively, indicating higher NHR was related to increased CVD mortality in the ageing population (**Table 2**). Every one SD increase in the NHR was linked to an increased risk of CVD mortality in the ageing population (HR = 1.12; 95% CI = 1.08–1.17) after adjusting for confounding factors. The RCS curves displayed a linear correlation (*P*-value for nonlinearity >0.05) between NHR and CVD mortality in the ageing population (**Figure 2**, Panel B).

**Table 2:** Associations between LHR, MHR, NHR, PHR, and CVD mortality in the ageing population*

	Model 1		Model 2		Model 3	
	**HR (95% CI)**	***P*-value**	**HR (95% CI)**	***P*-value**	**HR (95% CI)**	***P*-value**
**LHR tertiles**						
*T1*	ref		ref		ref	
*T2*	0.86 (0.70–1.07)	0.175	0.92 (0.75–1.14)	0.451	0.78 (0.62–0.98)	0.037
*T3*	0.79 (0.62–1.02)	0.067	0.92 (0.72–1.19)	0.527	0.69 (0.53–0.90)	0.007
*P–value for trend*		0.063		0.504		0.006
**MHR tertiles**						
*T1*	ref		ref		ref	
*T2*	1.46 (1.14–1.88)	0.003	1.38 (1.09–1.76)	0.008	1.21 (0.94–1.54)	0.133
*T3*	1.90 (1.47–2.46)	<0.001	1.44 (1.11–1.86)	0.006	1.07 (0.82–1.40)	0.625
*P–value for trend*		<0.001		0.008		0.717
**NHR tertiles**						
*T1*	ref		ref		ref	
*T2*	1.41 (1.05–1.88)	0.021	1.27 (0.97–1.67)	0.081	1.10 (0.82–1.47)	0.515
*T3*	2.51 (1.91–3.32)	<0.001	2.09 (1.58–2.77)	<0.001	1.70 (1.23–2.36)	0.001
*P–value for trend*		<0.001		<0.001		<0.001
**PHR tertiles**						
*T1*	ref		ref		ref	
*T2*	0.93 (0.74–1.17)	0.551	1.03 (0.83–1.28)	0.796	0.95 (0.76 1.19)	0.646
*T3*	0.96 (0.74–1.24)	0.728	1.08 (0.86–1.36)	0.520	0.94 (0.72–1.22)	0.653
*P–value for trend*		0.744		0.522		0.659

CI – confidence interval, HR – hazard ratio, LHR – lymphocyte/high density lipoprotein ratio, MHR – monocyte/high density lipoprotein ratio, NHR – neutrophil/high density lipoprotein ratio, PHR – platelet/high density lipoprotein ratio

*Model 1: unadjusted. Model 2: adjusted for age, gender, race, and BMI. Model 3: adjusted for age, gender, race, education level, family income-poverty ratio, smoking status, drinking status, BMI, TC TG, HbA1c, eGFR, hypertension, diabetes, and CVDs.

Similar to all-cause mortality, Kaplan-Meier survival curves (**Figure 2**, Panel C) showed that increased LHR was linked to decreased CVD mortality (log-rank *P* < 0.001). LHR did not present significant association with CVD mortality in model 1 and model 2. In adjusted model 3, however, decreased LHR was related to higher CVD mortality in the ageing population. For CVD mortality, using the lowest tertile (T1) as the reference, the HRs in the T2 and T3 groups were 0.78 (95% CI = 0.62–0.98) and 0.69 (95% CI = 0.53–0.90), respectively (*P*-value for trend = 0.006). LHR was not significantly associated with CVD mortality when analysed as a continuous variable. Correlation between LHR and CVD mortality was nonlinear (*P*-value for nonlinearity <0.0001) (**Figure 2**, Panel D). In the ageing population, neither MHR nor PHR was associated with CVD mortality.

### Subgroup analysis

**Figure 3:**
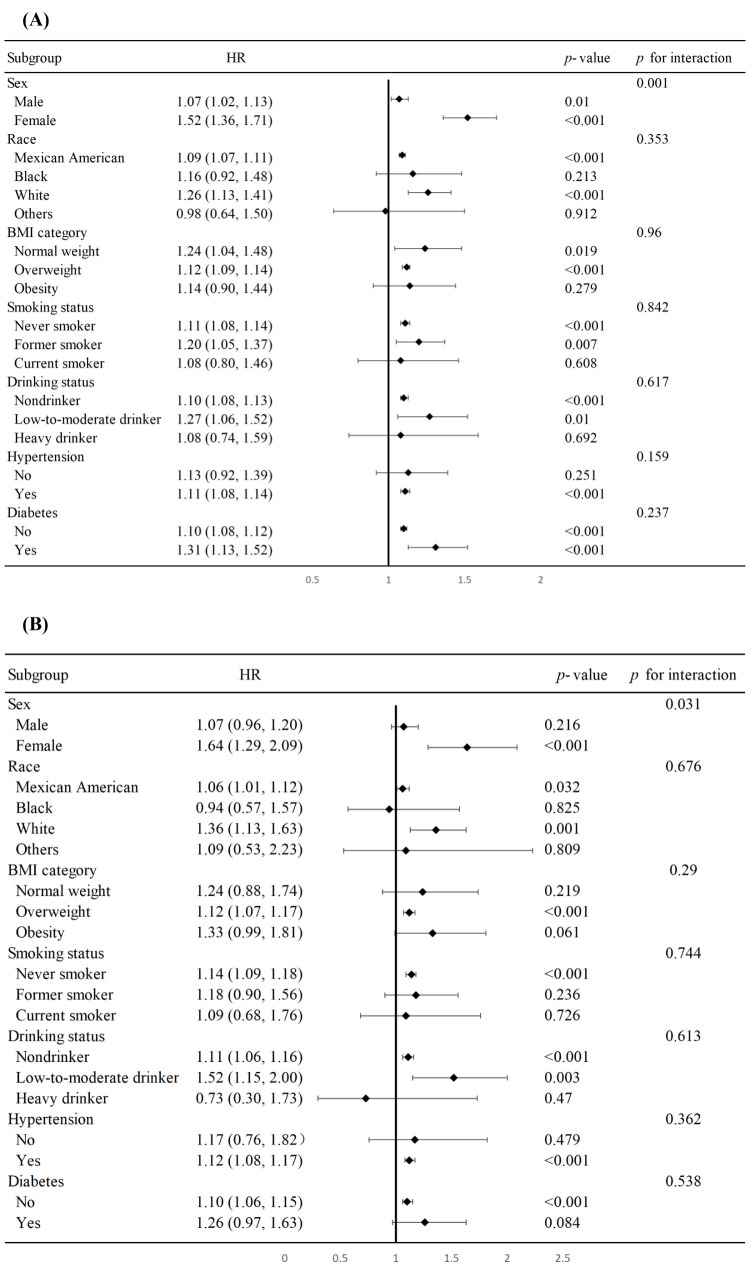
Subgroup analysis of the associations between the NHR and all-cause (**Panel A**) and CVD mortality (**Panel B**) in the ageing population.

We noted a correlation of NHR with all-cause mortality in the ageing population among participants who were male, female, Mexican American, White, normal weight, overweight, never smokers, former smokers, non-drinkers, and low-to-moderate drinkers, as well as individuals with hypertension and with or without diabetes (**Figure 3**, Panel A). CVD mortality was elevated among participants who were female, Mexican American, White, overweight, never smokers, non-drinkers, and low-to-moderate drinkers, and individuals with hypertension or without diabetes. Furthermore, in gender subgroups, interactions between NHR and both all-cause and CVD mortality in the ageing population demonstrated a more pronounced effect among females (**Figure 3**, Panel B).

### The prognostic ability of NHR for all-cause and CVD mortality

The addition of NHR to the basic model was associated with a modest improvement in discrimination for all-cause mortality (C-statistic increase, *P* = 0.023). We observed on significant improvement for CVD mortality (**Table 3**). When LHR was analysed as a continuous variable, it exhibited no significant association with all-cause and CVD mortality. Therefore, we did not present the C-statistic of LHR.

**Table 3:** Improvement in discrimination and risk reclassification for all-cause and CVD mortality after NHR

	C-statistic (95% CI)	*P*-value
**All-cause mortality**		
Basic model	0.758 (0.746–0.770)	ref
Basic model + NHR	0.769 (0.748–0.772)	0.023
**CVD mortality**		
Basic model	0.751 (0.739–0.753)	ref
Basic model + NHR	0.753 (0.741–0.765)	0.287

CI – confidence interval, CVD – cardiovascular disease, NHR – neutrophil/high density lipoprotein ratio, ref – reference

Basic model was adjusted for age, gender, race, education level, family income poverty ratio, smoking status, drinking status, BMI, TC TG, HbA1c, eGFR, hypertension, diabetes, and CVD.

## DISCUSSION

In this study, we used data on 5700 participants from the NHANES 2001–2018 cycles to explore HDL-related inflammatory markers with all-cause and CVD mortality. We found that higher NHR, whether analysed as a continuous or categorical variable, was linked to elevated all-cause and CVD mortality. In contrast, higher LHR was linked to decreased mortality, but only when analysed as a categorical variable. The association of MHR and PHR with all-cause and CVD mortality was non-significant. An RCS analysis further confirmed the association between NHR and both all-cause and CVD mortality, and showed a non-linear correlation between LHR and both mortality outcomes. We note, however, that the inverse association between LHR and mortality may reflect confounding by overall health status, frailty, or reverse causation, rather than a direct protective effect. Overall, our findings highlight the association of NHR with all-cause mortality in the ageing population.

Lipid metabolism and inflammation are closely intertwined in the development of CVDs, as they influence one another bidirectionally. Dysregulation of lipid metabolism, particularly abnormalities in cholesterol and TG levels, can trigger and exacerbate inflammation, which in turn accelerates the pathogenesis of atherosclerosis and other cardiovascular conditions [24,25]. HDL-C is considered beneficial for cardiovascular health because it removes surplus cholesterol from arterial walls and transports it to the liver for excretion. HDL also demonstrates anti-inflammatory effects by decreasing the activation of immune cells and promoting the resolution of inflammation [26]. Low HDL levels are related to increased inflammation and higher cardiovascular risk [27]. LHR, MHR, NHR, and PHR are HDL-related inflammatory markers, providing comprehensive information of lipid metabolism and inflammation in a variety of diseases [9].

In prior research, NHR levels have been significantly and positively correlated with biochemical indicators of myocardial ischaemia and remodelling in type 2 diabetes mellitus and acute coronary syndrome patients [28,29]. NHR has also been associated with CAD, serving as a better predictor of severe CAD than neutrophil and HDL-C [30]. High NHR has been associated with worse survival outcomes in liver cancer [31], acute biliary pancreatitis [32], Parkinson’s disease [33], and periodontitis [34]. Here, we noted that an increased NHR was related to higher all-cause and CVD mortality, and observed a linear relationship between NHR and mortality in the ageing population based on survey-weighted Cox regression analysis. This is in contrast with research among the general population, where the relation between NHR and all-cause mortality was found to be nonlinear [35]. This may be explained by different concentrations of HDL among participants in our cohort.

Studies related to LHR have arrived at contradictory conclusions in different diseases. Specifically, LHR was found to be a useful prognostic marker in sepsis patients, with an LHR ≤0.6 indicating a harmful burden that raises mortality risk [36]. In chronic obstructive pulmonary disease patients, a reduced LHR was independently linked to worse pulmonary function [37]. Conversely, a higher level of LHR was associated with higher depression morbidity in adults from the USA [38] and acted as a predictor for metabolic syndrome [39]. However, the potential of LHR as a prognostic indicator for mortality is yet to be fully examined. In our study, the association between LHR and mortality was nonlinear. The significant findings in tertile analyses likely reflect this curved dose, response relationship, where lower LHR values are more strongly inversely associated with mortality risk. For MHR and PHR, there were not significant association with all-cause and CVD mortality.

The associations of LHR and NHR with prognosis likely reflect distinct immune and inflammatory pathways, with the former reflecting adaptive immunity and the latter reflecting innate immunity. We hypothesise that lower LHR may be a marker of poorer general health or immune competence, while higher NHR indicates a pro-inflammatory state. Lymphocytes are a crucial component of the adaptive immune system, responsible for recognising and responding to specific pathogens, tumours, and abnormal cells, and contributing to long-term immune surveillance and defence [40]. A decrease in lymphocyte count can be indicative of immune suppression, immune system exhaustion, or an impaired adaptive immune response [41,42]. A lower lymphocyte count is a well-documented marker of immunosuppression, chronic inflammation, or immune system dysfunction [43]. In CVDs, a lower LHR is often seen in patients with lipid metabolism disturbances and a reduced immune response, contributing to poor cardiovascular outcomes [44]. Neutrophils contribute to plaque rupture, thrombosis, and vessel damage [45], while low HDL reduces the ability to clear cholesterol from the arteries, exacerbating plaque buildup.

Higher NHR reflects an imbalance between the pro-inflammatory state and the anti-inflammatory, protective effect of HDL, and the inability to efficiently resolve inflammation and promote vascular repair contributes to plaque instability and cardiovascular events.

We noted a correlation of NHR with mortality in males and females in the ageing population which was more pronounced among the latter group. This can be attributed to several biological, hormonal, and immune system differences that affect the way inflammation, lipid metabolism, and cardiovascular risk manifest in males and females as they age [46]. In general, females tend to have a more active immune system compared to males, which is driven by sex hormones, but particularly oestrogen, which has immunomodulatory effects that enhance immune responses, including the activation of neutrophils and the expression of pro-inflammatory cytokines [47]. The hormonal changes that occur with menopause, especially declines in oestrogen, also lead to a reduction in HDL cholesterol levels [48]. The dysregulation of both lipid metabolism and immune responses works together to exacerbate the risk of CVD and mortality [49]. Therefore, these changes in females are more pronounced as they age, leading to a stronger link between NHR and mortality, resulting in a more significant risk of CVD mortality in females, making NHR a stronger predictor of poor outcomes in ageing females compared to males. We note, however, that this and other subgroup findings in our study are exploratory; they should not be interpreted as confirmatory of mechanistic differences.

The strengths of our study include a representative, high-quality dataset, a long follow-up duration, and sampling weighting, all of which increase the robustness of our findings. Nevertheless, it also has some limitations. First, as an observational study with a single baseline measurement, causal inference is limited and reverse causation cannot be excluded. Second, while we adjusted for major confounders, residual confounding from unmeasured factors (*e.g.* medication use, frailty, physical activity) remains possible. Third, the lack of repeated measurements precluded us from assessing marker dynamics over time. Fourth, our broad definition of CVD mortality and the use of tertiles based on the pooled sample may have obscured subtype-specific associations and could have been influenced by temporal changes in diagnostic criteria, treatment practices, or data collection over the 18-year study period. Fifth, covariates based on self-reported data are subject to recall bias. Finally, we did not perform sensitivity analyses such as excluding early deaths or prevalent CVD; this choice, while supporting generalisability, may have increased susceptibility to reverse causality. Therefore, our findings should be interpreted as potential correlational, rather than causative associations.

## CONCLUSIONS

This study identified a potential correlation between the HDL-related inflammatory markers and both all-cause and CVD mortality in the ageing population, with heterogeneity across the aforementioned markers. Higher NHR was linearly associated with poorer outcomes, while lower LHR was associated with higher mortality, though this may be influenced by health status and reverse causation. PHR and MHR were not significantly related with all-cause and CVD outcomes. While NHR was significantly associated with mortality risk in the ageing population, we note that NHANES is not designed as a clinical risk-prediction cohort, so our findings require further validation. The divergent patterns observed for other markers highlight that they may reflect different underlying biological pathways. Future research should focus on validating the prognostic utility of NHR and elucidating the distinct mechanisms represented by these related, but heterogeneous inflammatory-lipid markers.

## Additional material


Online Supplementary Document
