# Supplementary material for: High-density lipoprotein-related inflammatory markers and their association with all-cause and cardiovascular mortality in an ageing population: findings from a prospective cohort study based on NHANES data
Source: J Glob Health. 2026 Mar 27;16:04107. doi: 10.7189/jogh.16.04107 (PMC13021054; doi:10.7189/jogh.16.04107)
Supplement: Online Supplementary Document [file jogh-16-04107-s001.pdf]

Supplement to: Hu T, Chen R, Gu Y, Yu C, Liu X, Zhang X. High-density lipoprotein-related inflammatory markers and their association with all-cause and cardiovascular mortality in an ageing population: findings from a prospective cohort study based on NHANES data. J Glob Health. 2026;16:04107.

Figure S1. Flow chart of the study participants.

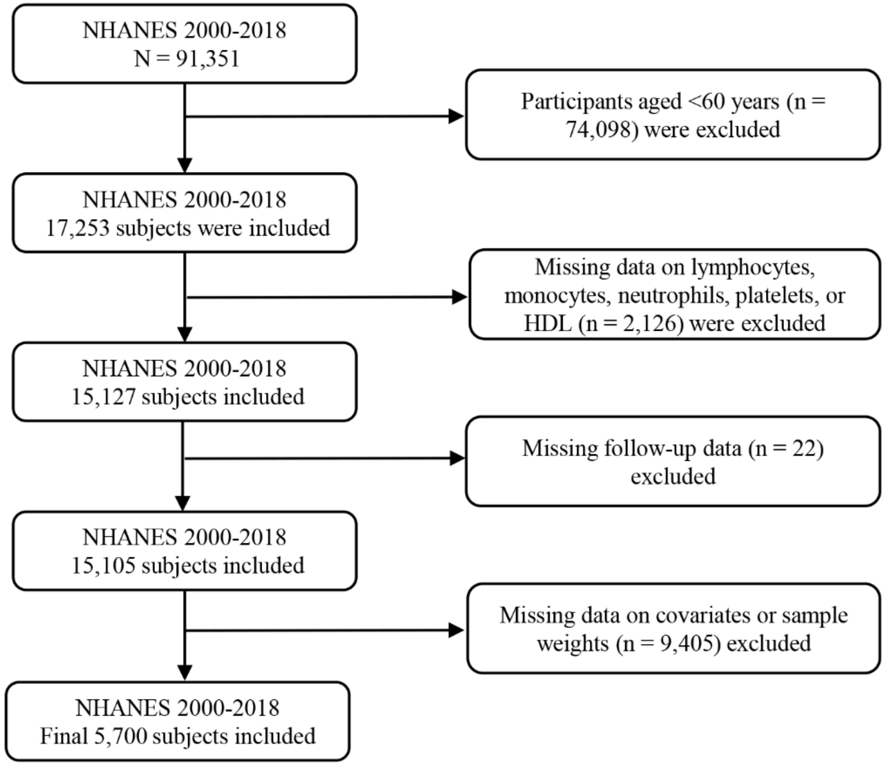

Table S1. Outline of JoGH's Guidelines for Reporting Analyses of Big Data Repositories Open to the Public (GRABDROP) items

1. Please list all papers published by each co-author in previous three years that were based on secondary analysis of a big data repository	None.
2. Please explain the key elements of your study design and the use of the available datasets that make your study an original scientific contribution	This study provides an original investigation into four novel HDL-related inflammatory ratios (NHR, LHR, MHR, PHR) and their associations with mortality, specifically within a nationally representative aging population (NHANES, age ≥60). Its contribution lies in the concurrent analysis and comparison of these emerging markers, the application of rigorous survey-weighted survival analysis, and the elucidation of their distinct linear (NHR) and non-linear (LHR) relationships with mortality outcomes in a real-world older cohort.
3. Please list all publications that addressed similar research questions in the same dataset and indicate where you cited them in your paper	REN H, ZHU B, ZHAO Z, et al. Neutrophil to high-density lipoprotein cholesterol ratio as the risk mark in patients with type 2 diabetes combined with acute coronary syndrome: a cross-sectional study [J]. Sci Rep, 2023, 13(1): 7836. DOI: 10.1038/s41598-023-35050-6. (Cited in Discussion)<br>DZIEDZIC EA, GĄSIOR JS, KOSESKA K, et al. The Impact of Neutrophil-to-High-Density Lipoprotein Ratio and Serum 25-Hydroxyvitamin D on Ischemic Heart Disease [J]. J Clin Med, 2024, 13(21). DOI: 10.3390/jcm13216597. (Cited in Discussion)<br>Our study distinguishes itself from these prior works in the same dataset by focusing specifically on the aging population (≥60 years) and investigating the comparative and differential associations of four related inflammatory-lipid markers (NHR, LHR, MHR, PHR) with both all-cause and cardiovascular mortality, rather than a single marker or a specific disease subgroup.
4. Please explain how you addressed multiple	We addressed multiple testing by pre-specifying the NHR as the sole primary exposure for

testing through an appropriately rigorous statistical threshold and indicate this in the methods section	hypothesis testing at P < 0.05. All other markers (LHR, MHR, PHR) and subgroup analyses were explicitly designated as exploratory and descriptive in the Methods, as noted in the "Statistical analysis" section, thereby controlling the type I error for the primary hypothesis while providing contextual findings.
5. Please declare to what extent have AI chatbots been used in developing your paper and to which parts of the paper did they contribute	No AI chatbots (ChatGPT or similar) were used in the conceptualization, study design, data analysis, interpretation of results, or drafting of this manuscript.

Table S2. Baseline characteristics of included participants stratified by survival status.

Characteristic	Total (n = 5,700)	Survivor (n = 3,883)	Non-survivors (n = 1,817)	*p*-value
**Age, years**	68 (64, 75)	67 (63, 72)	75 (68, 80)	<0.001
**Sex**				<0.001
Male	2,853 (45.6%)	1,821 (43.7%)	1,032 (50.7%)	
Female	2,847 (54.4%)	2,062 (56.3%)	785 (49.3%)	
**Race**				<0.001
Mexican American	781 (3.8%)	593 (4.2%)	188 (2.8%)	
Black	998 (8.2%)	729 (8.3%)	269 (7.8%)	
White	3,084 (79.5%)	1,843 (77.8%)	1,241 (84.3%)	
Others	837 (8.4%)	718 (9.7%)	119 (5.1%)	
**Education levels**				<0.001
Under high school	952 (8.8%)	588 (7.1%)	364 (13.6%)	
High school or equivalent	2,195 (36.8%)	1,407 (33.7%)	788 (45.1%)	
Above high school	2,553 (54.4%)	1,888 (59.2%)	665 (41.3%)	
**Family income poverty ratio**				<0.001
<1	917 (9.1%)	615 (8.2%)	302 (11.5%)	
1-3	2,702 (41.2%)	1,714 (36.7%)	988 (53.3%)	
≥3	2,081 (49.7%)	1,554 (55.1%)	527 (35.2%)	
**Marital status**				<0.001
Married or living with a partner	3,465 (65.1%)	2,502 (68.8%)	963 (55.2%)	

Characteristic	Total (n = 5,700)	Survivor (n = 3,883)	Non-survivors (n = 1,817)	*p*-value
Others	2,235 (34.9%)	1,381 (31.2%)	854 (44.8%)	
**Smoking status**				<0.001
Never smoker	2,740 (47.8%)	2,000 (50.8%)	740 (39.6%)	
Former smoker	2,276 (41.1%)	1,453 (39.1%)	823 (46.5%)	
Current smoker	684 (11.1%)	430 (10.0%)	254 (13.9%)	
**Drinking status**				<0.001
Nondrinker	2,719 (41.6%)	1,727 (37.7%)	992 (52.1%)	
Low-to-moderate drinker	2,113 (42.4%)	1,519 (45.3%)	594 (34.5%)	
Heavy drinker	868 (16.0%)	637 (17.0%)	231 (13.3%)	
**BMI, kg/m^2**	27.9 (24.7, 32.1)	28.2 (25.1, 32.3)	27.3 (24.0, 31.3)	<0.001
**BMI category**				<0.001
Normal weight (<25)	1,532 (26.8%)	931 (24.6%)	601 (32.6%)	
Overweight (25-30)	2,099 (36.2%)	1,447 (36.7%)	652 (34.8%)	
Obesity (≥30)	2,069 (37.0%)	1,505 (38.7%)	564 (32.7%)	
**TC, mmol/L**	4.97 (4.27, 5.74)	5.02 (4.32, 5.77)	4.91 (4.19, 5.64)	0.004
**TG, mmol/L**	1.26 (0.90, 1.78)	1.24 (0.88, 1.75)	1.34 (0.97, 1.91)	<0.001
**LDL, mmol/L**	2.87 (2.25, 3.49)	2.90 (2.25, 3.54)	2.77 (2.17, 3.41)	<0.001
**HDL, mmol/L**	1.40 (1.14, 1.73)	1.40 (1.16, 1.76)	1.32 (1.11, 1.66)	<0.001
**HbA1c, %**	5.70 (5.40, 6.10)	5.70 (5.40, 6.00)	5.70 (5.40, 6.10)	0.3
**Fast glucose, mmol/L**	5.83 (5.38, 6.55)	5.83 (5.38, 6.50)	5.83 (5.33, 6.72)	0.8
**Serum creatinine, mg/dl**	0.91 (0.78, 1.09)	0.90 (0.76, 1.05)	0.99 (0.80, 1.20)	<0.001

Characteristic	**Total**<br>(n = 5,700)	**Survivor**<br>(n = 3,883)	**Non-survivors**<br>(n = 1,817)	***p*-value**
**eGFR, ml/min/1.73m^2**	78.5 (65.0, 91.6)	81.1 (68.3, 93.5)	71.8 (56.6, 86.6)	<0.001
**Lymphocytes, ×10^9/L**	1.70 (1.40, 2.10)	1.80 (1.40, 2.20)	1.70 (1.30, 2.10)	<0.001
**Monocytes, ×10^9/L**	0.50 (0.40, 0.70)	0.50 (0.40, 0.60)	0.60 (0.50, 0.70)	<0.001
**Neutrophils, ×10^9/L**	3.70 (2.90, 4.70)	3.60 (2.90, 4.60)	4.00 (3.20, 5.20)	<0.001
**Platelets, ×10^9/L**	228 (191, 270)	228 (192, 268)	229 (186, 274)	>0.9
**LHR**	1.23 (0.89, 1.67)	1.23 (0.90, 1.68)	1.21 (0.85, 1.65)	0.021
**MHR**	0.38 (0.28, 0.53)	0.37 (0.27, 0.51)	0.40 (0.30, 0.57)	<0.001
**NHR**	2.67 (1.86, 3.78)	2.55 (1.77, 3.62)	2.99 (2.09, 4.14)	<0.001
**PHR**	162.4 (126.1, 206.8)	160.6 (124.9, 203.8)	167.9 (127.9, 213.4)	0.007
**Hypertension**	4,020 (67.5%)	2,646 (64.7%)	1,374 (75.1%)	<0.001
**Diabetes**	1,957 (30.0%)	1,311 (27.9%)	646 (35.5%)	<0.001
**CVD**	1,380 (23.7%)	753 (19.3%)	627 (35.7%)	<0.001

Table S3. Baseline characteristics of the study population stratified by NHR tertiles.

Characteristic	Total (n =5,700)	< 2.18 (n =1,902)	2.18-3.37 (n =1,898)	> 3.37 (n =1,900)	*p*-value
**Age, years**	68 (64, 75)	68 (63, 74)	69 (64, 76)	69 (64, 76)	0.019
**Sex**					<0.001
Male	2,853 (45.6%)	688 (31.0%)	939.0 (46.3%)	1,226.0 (60.7%)	
Female	2,847 (54.4%)	1,214 (69.0%)	959.0 (53.7%)	674.0 (39.3%)	
**Race**					<0.001
Mexican American	781 (3.8%)	196 (3.0%)	266 (3.9%)	319 (4.7%)	
Black	998 (8.2%)	505 (12.0%)	297 (7.4%)	196 (4.8%)	
White	3,084 (79.5%)	917 (76.6%)	1,036 (79.7%)	1,131 (82.6%)	
Others	837 (8.4%)	284 (8.4%)	299 (9.0%)	254 (7.9%)	
**Education levels**					<0.001
Under high school	952 (8.8%)	254 (6.7%)	333 (9.6%)	365 (10.5%)	
High school or equivalent	2,195 (36.8%)	694 (33.3%)	711 (37.1%)	790 (40.2%)	
Above high school	2,553 (54.4%)	954 (60.0%)	854 (53.3%)	745 (49.3%)	
**Family income poverty ratio**					<0.001
<1	917 (9.1%)	276 (7.9%)	285 (8.2%)	356 (11.3%)	
1-3	2,702 (41.2%)	840 (36.3%)	922 (42.8%)	940 (44.8%)	
≥3	2,081 (49.7%)	786 (55.7%)	691 (49.0%)	604 (43.9%)	
**Marital status**					0.9
Married or living with a partner	3,465 (65.1%)	1,128 (64.8%)	1,169 (64.9%)	1,168 (65.7%)	

**Characteristic**	**Total**<br>(n =5,700)	**< 2.18**<br>(n =1,902)	**2.18-3.37**<br>(n =1,898)	**> 3.37**<br>(n =1,900)	***p*-value**
Others	2,235 (34.9%)	774 (35.2%)	729 (35.1%)	732 (34.3%)	
**Smoking status**					<0.001
Never smoker	2,740 (47.8%)	1,068 (54.8%)	968 (50.8%)	704 (37.0%)	
Former smoker	2,276 (41.1%)	693 (39.5%)	748 (40.5%)	835 (43.6%)	
Current smoker	684 (11.1%)	141 (5.6%)	182 (8.7%)	361 (19.5%)	
**Drinking status**					<0.001
Nondrinker	2,719 (41.6%)	804 (34.3%)	939 (44.3%)	976 (46.9%)	
Low-to-moderate drinker	2,113 (42.4%)	767 (45.6%)	677 (40.9%)	669 (40.4%)	
Heavy drinker	868 (16.0%)	331 (20.1%)	282 (14.8%)	255 (12.7%)	
**BMI, kg/m^2**	27.9 (24.7, 32.1)	26.4 (23.7, 29.6)	28.5 (25.1, 32.7)	29.8 (26.2, 34.1)	<0.001
**BMI category**					<0.001
Normal weight (<25)	1,532 (26.8%)	687 (37.5%)	474 (24.3%)	371 (17.6%)	
Overweight (25-30)	2,099 (36.2%)	710 (39.0%)	699 (35.5%)	690 (33.8%)	
Obesity (≥30)	2,069 (37.0%)	505 (23.5%)	725 (40.1%)	839 (48.6%)	
**TC, mmol/L**	4.97 (4.27, 5.74)	5.33 (4.65, 6.03)	4.97 (4.27, 5.69)	4.60 (3.90, 5.30)	<0.001
**TG, mmol/L**	1.26 (0.90, 1.78)	0.99 (0.76, 1.32)	1.33 (0.96, 1.77)	1.63 (1.15, 2.18)	<0.001
**LDL, mmol/L**	2.87 (2.25, 3.49)	2.97 (2.41, 3.62)	2.90 (2.28, 3.54)	2.66 (2.02, 3.26)	<0.001
**HDL, mmol/L**	1.40 (1.14, 1.73)	1.78 (1.50, 2.07)	1.34 (1.22, 1.58)	1.11 (0.98, 1.29)	<0.001
**HbA1c, %**	5.70 (5.40, 6.10)	5.60 (5.30, 5.90)	5.70 (5.40, 6.10)	5.80 (5.50, 6.40)	<0.001
**Fast glucose, mmol/L**	5.83 (5.38, 6.55)	5.55 (5.20, 6.10)	5.88 (5.44, 6.55)	6.22 (5.66, 7.22)	<0.001
**Serum creatinine, mg/dl**	0.91 (0.78, 1.09)	0.85 (0.72, 1.00)	0.92 (0.80, 1.09)	0.98 (0.81, 1.17)	<0.001

Characteristic	Total<br>(n =5,700)	< 2.18<br>(n =1,902)	2.18-3.37<br>(n =1,898)	> 3.37<br>(n =1,900)	*p*-value
**eGFR, ml/min/1.73m^2**	78.5 (65.0, 91.6)	81.5 (67.9, 93.6)	78.0 (65.0, 90.5)	76.4 (62.7, 89.9)	<0.001
**Lymphocytes, ×10^9/L**	1.70 (1.40, 2.10)	1.60 (1.40, 2.00)	1.70 (1.40, 2.20)	1.80 (1.50, 2.30)	<0.001
**Monocytes, ×10^9/L**	0.50 (0.40, 0.70)	0.50 (0.40, 0.60)	0.50 (0.40, 0.60)	0.60 (0.50, 0.80)	<0.001
**Neutrophils, ×10^9/L**	3.70 (2.90, 4.70)	2.80 (2.30, 3.30)	3.70 (3.30, 4.30)	5.10 (4.40, 6.00)	<0.001
**Platelets, ×10^9/L**	228 (191, 270)	224 (187, 260)	227 (191, 265)	236 (194, 282)	<0.001
**LHR**	1.23 (0.89, 1.67)	0.92 (0.72, 1.20)	1.24 (0.97, 1.64)	1.63 (1.25, 2.14)	<0.001
**MHR**	0.38 (0.28, 0.53)	0.26 (0.21, 0.34)	0.39 (0.31, 0.48)	0.55 (0.43, 0.70)	<0.001
**NHR**	2.67 (1.86, 3.78)	1.63 (1.29, 1.89)	2.71 (2.45, 2.99)	4.40 (3.81, 5.41)	<0.001
**PHR**	162.4 (126.1, 206.8)	126.8 (100.0, 157.2)	163.7 (136.7, 198.4)	208.2 (168.8, 255.2)	<0.001
**Hypertension**	4,020 (67.5%)	1,250 (59.4%)	1,361 (69.4%)	1,409 (74.5%)	<0.001
**Diabetes**	1,957 (30.0%)	447 (17.3%)	650 (30.7%)	860 (43.0%)	<0.001
**CVD**	1,380 (23.7%)	314 (16.2%)	447 (22.6%)	619 (33.1%)	<0.001

Table S4. Baseline characteristics of the study population stratified by LHR tertiles.

Characteristic	Total (n =5,700)	< 1.05 (n =1,903)	1.05-1.57 (n =1,897)	> 1.57 (n =1,900)	*p*-value
**Age, years**	68 (64, 75)	70 (64, 77)	69 (64, 75)	67 (63, 73)	<0.001
**Sex**					<0.001
Male	2,853 (45.6%)	823 (38.8%)	930 (44.3%)	1,100 (55.1%)	
Female	2,847 (54.4%)	1,080 (61.2%)	967 (55.7%)	800 (44.9%)	
**Race**					<0.001
Mexican American	781 (3.8%)	165 (2.2%)	296 (4.7%)	320 (4.9%)	
Black	998 (8.2%)	341 (7.9%)	317 (7.8%)	340 (8.9%)	
White	3,084 (79.5%)	1,170 (83.9%)	1,010 (79.2%)	904 (74.7%)	
Others	837 (8.4%)	227 (6.0%)	274 (8.4%)	336 (11.4%)	
**Education levels**					<0.001
Under high school	952 (8.8%)	239 (6.3%)	306 (8.8%)	407 (11.9%)	
High school or equivalent	2,195 (36.8%)	673 (32.7%)	764 (39.5%)	758 (38.9%)	
Above high school	2,553 (54.4%)	991 (61.1%)	827 (51.7%)	735 (49.2%)	
**Family income poverty ratio**					<0.001
<1	917 (9.1%)	245 (6.9%)	297 (9.4%)	375 (11.4%)	
1-3	2,702 (41.2%)	876 (38.2%)	901 (42.9%)	925 (42.9%)	
≥3	2,081 (49.7%)	782 (54.9%)	699 (47.7%)	600 (45.6%)	
**Marital status**					0.5
Married or living with a partner	3,465 (65.1%)	1,086 (63.6%)	1,184 (65.6%)	1,195 (66.3%)	

**Characteristic**	**Total** (n =5,700)	**< 1.05** (n =1,903)	**1.05-1.57** (n =1,897)	**> 1.57** (n =1,900)	***p*-value**
Others	2,235 (34.9%)	817 (36.4%)	713 (34.4%)	705 (33.7%)	
**Smoking status**					<0.001
Never smoker	2,740 (47.8%)	978 (51.3%)	950 (48.4%)	812 (42.9%)	
Former smoker	2,276 (41.1%)	786 (43.1%)	734 (40.6%)	756 (39.4%)	
Current smoker	684 (11.1%)	139 (5.6%)	213 (11.0%)	332 (17.6%)	
**Drinking status**					0.002
Nondrinker	2,719 (41.6%)	828 (37.4%)	893 (42.1%)	998 (46.2%)	
Low-to-moderate drinker	2,113 (42.4%)	775 (45.2%)	718 (42.6%)	620 (38.7%)	
Heavy drinker	868 (16.0%)	300 (17.4%)	286 (15.2%)	282 (15.1%)	
**BMI, kg/m^2**	27.9 (24.7, 32.1)	26.2 (23.5, 29.9)	28.8 (25.4, 33.2)	29.4 (26.2, 33.4)	<0.001
**BMI category**					<0.001
Normal weight (<25)	1,532 (26.8%)	732 (39.3%)	456 (22.0%)	344 (16.8%)	
Overweight (25-30)	2,099 (36.2%)	672 (36.0%)	717 (36.4%)	710 (36.2%)	
Obesity (≥30)	2,069 (37.0%)	499 (24.7%)	724 (41.6%)	846 (47.0%)	
**TC, mmol/L**	4.97 (4.27, 5.74)	5.20 (4.50, 5.90)	4.94 (4.22, 5.66)	4.76 (4.11, 5.51)	<0.001
**TG, mmol/L**	1.26 (0.90, 1.78)	0.98 (0.73, 1.33)	1.30 (0.95, 1.81)	1.67 (1.22, 2.19)	<0.001
**LDL, mmol/L**	2.87 (2.25, 3.49)	2.87 (2.28, 3.47)	2.90 (2.25, 3.52)	2.82 (2.17, 3.49)	0.3
**HDL, mmol/L**	1.40 (1.14, 1.73)	1.76 (1.47, 2.04)	1.37 (1.19, 1.58)	1.11 (0.98, 1.29)	<0.001
**HbA1c, %**	5.70 (5.40, 6.10)	5.60 (5.30, 5.80)	5.70 (5.50, 6.10)	5.80 (5.50, 6.30)	<0.001
**Fast glucose, mmol/L**	5.83 (5.38, 6.55)	5.63 (5.22, 6.16)	5.88 (5.38, 6.61)	6.16 (5.61, 7.05)	<0.001
**Serum creatinine, mg/dl**	0.91 (0.78, 1.09)	0.90 (0.76, 1.07)	0.90 (0.78, 1.08)	0.95 (0.80, 1.10)	<0.001

Characteristic	**Total** (n =5,700)	**< 1.05** (n =1,903)	**1.05-1.57** (n =1,897)	**> 1.57** (n =1,900)	***p*-value**
**eGFR, ml/min/1.73m^2**	78.5 (65.0, 91.6)	78.0 (64.8, 91.2)	79.0 (66.3, 91.3)	78.7 (64.2, 93.3)	0.8
**Lymphocytes, ×10^9/L**	1.70 (1.40, 2.10)	1.40 (1.10, 1.60)	1.80 (1.50, 2.00)	2.30 (2.00, 2.70)	<0.001
**Monocytes, ×10^9/L**	0.50 (0.40, 0.70)	0.50 (0.40, 0.60)	0.60 (0.40, 0.70)	0.60 (0.50, 0.70)	<0.001
**Neutrophils, ×10^9/L**	3.70 (2.90, 4.70)	3.40 (2.70, 4.20)	3.70 (3.00, 4.80)	4.10 (3.40, 5.20)	<0.001
**Platelets, ×10^9/L**	228 (191, 270)	220 (182, 258)	229 (193, 271)	236 (200, 282)	<0.001
**LHR**	1.23 (0.89, 1.67)	0.81 (0.65, 0.93)	1.27 (1.14, 1.42)	1.97 (1.72, 2.31)	<0.001
**MHR**	0.38 (0.28, 0.53)	0.28 (0.22, 0.36)	0.39 (0.30, 0.50)	0.54 (0.42, 0.68)	<0.001
**NHR**	2.67 (1.86, 3.78)	1.93 (1.45, 2.68)	2.75 (2.07, 3.66)	3.71 (2.73, 4.91)	<0.001
**PHR**	162.4 (126.1, 206.8)	126.2 (100.0, 157.8)	165.0 (138.6, 198.8)	209.3 (171.7, 254.7)	<0.001
**Hypertension**	4,020 (67.5%)	1,277 (61.6%)	1,371 (70.2%)	1,372 (71.7%)	<0.001
**Diabetes**	1,957 (30.0%)	484 (20.3%)	630 (30.6%)	843 (40.9%)	<0.001
**CVD**	1,380 (23.7%)	419 (19.8%)	457 (24.8%)	504 (27.3%)	<0.001

Table S5. Baseline characteristics of the study population stratified by MHR tertiles.

**Characteristic**	**Total** (n =5,700)	**< 0.31** (n =1,909)	**0.31-0.47** (n =1,897)	**> 0.47** (n =1,894)	***p*-value**
**Age, years**	68 (64, 75)	68 (63, 75)	68 (64, 75)	69 (64, 76)	0.2
**Sex**					<0.001
Male	2,853 (45.6%)	614 (27.8%)	917 (43.7%)	1,322 (65.9%)	
Female	2,847 (54.4%)	1,295 (72.2%)	980 (56.3%)	572 (34.1%)	
**Race**					<0.001
Mexican American	781 (3.8%)	240 (3.5%)	289 (4.4%)	252 (3.7%)	
Black	998 (8.2%)	443 (10.9%)	316 (7.8%)	239 (5.7%)	
White	3,084 (79.5%)	925 (77.0%)	1,017 (79.7%)	1,142 (82.1%)	
Others	837 (8.4%)	301 (8.7%)	275 (8.2%)	261 (8.5%)	
**Education levels**					<0.001
Under high school	952 (8.8%)	284 (7.7%)	321 (8.9%)	347 (10.0%)	
High school or equivalent	2,195 (36.8%)	688 (33.2%)	729 (36.8%)	778 (40.5%)	
Above high school	2,553 (54.4%)	937 (59.1%)	847 (54.3%)	769 (49.5%)	
**Family income poverty ratio**					0.004
<1	917 (9.1%)	278 (7.9%)	308 (9.5%)	331 (10.0%)	
1-3	2,702 (41.2%)	857 (38.1%)	907 (42.4%)	938 (43.2%)	
≥3	2,081 (49.7%)	774 (54.0%)	682 (48.2%)	625 (46.8%)	
**Marital status**					0.066
Married or living with a partner	3,465 (65.1%)	1,108 (63.9%)	1,153 (63.5%)	1,204 (67.9%)	

**Characteristic**	**Total** (n =5,700)	**< 0.31** (n =1,909)	**0.31-0.47** (n =1,897)	**> 0.47** (n =1,894)	***p*-value**
Others	2,235 (34.9%)	801 (36.1%)	744 (36.5%)	690 (32.1%)	
**Smoking status**					<0.001
Never smoker	2,740 (47.8%)	1,091 (54.9%)	931 (49.3%)	718 (38.9%)	
Former smoker	2,276 (41.1%)	660 (38.7%)	759 (40.3%)	857 (44.5%)	
Current smoker	684 (11.1%)	158 (6.4%)	207 (10.4%)	319 (16.6%)	
**Drinking status**					0.009
Nondrinker	2,719 (41.6%)	863 (37.4%)	905 (42.6%)	951.0 (45.0%)	
Low-to-moderate drinker	2,113 (42.4%)	733 (44.4%)	678 (41.0%)	702.0 (41.6%)	
Heavy drinker	868 (16.0%)	313 (18.1%)	314 (16.3%)	241.0 (13.4%)	
**BMI, kg/m^2**	27.9 (24.7, 32.1)	26.4 (23.6, 30.1)	28.1 (25.0, 32.0)	29.6 (26.2, 33.8)	<0.001
**BMI category**					<0.001
Normal weight (<25)	1,532 (26.8%)	685.0 (37.4%)	478.0 (24.8%)	369.0 (17.8%)	
Overweight (25-30)	2,099 (36.2%)	687.0 (37.3%)	719.0 (37.2%)	693.0 (34.0%)	
Obesity (≥30)	2,069 (37.0%)	537.0 (25.3%)	700.0 (38.1%)	832.0 (48.2%)	
**TC, mmol/L**	4.97 (4.27, 5.74)	5.33 (4.65, 6.03)	4.94 (4.24, 5.66)	4.63 (3.90, 5.35)	<0.001
**TG, mmol/L**	1.26 (0.90, 1.78)	1.02 (0.77, 1.40)	1.29 (0.91, 1.75)	1.58 (1.13, 2.16)	<0.001
**LDL, mmol/L**	2.87 (2.25, 3.49)	2.95 (2.38, 3.60)	2.87 (2.25, 3.49)	2.72 (2.07, 3.39)	<0.001
**HDL, mmol/L**	1.40 (1.14, 1.73)	1.78 (1.47, 2.07)	1.40 (1.22, 1.60)	1.11 (0.98, 1.27)	<0.001
**HbA1c, %**	5.70 (5.40, 6.10)	5.60 (5.30, 5.90)	5.70 (5.40, 6.10)	5.80 (5.50, 6.30)	<0.001
**Fast glucose, mmol/L**	5.83 (5.38, 6.55)	5.61 (5.22, 6.22)	5.83 (5.38, 6.44)	6.16 (5.61, 6.99)	<0.001
**Serum creatinine, mg/dl**	0.91 (0.78, 1.09)	0.84 (0.71, 1.00)	0.90 (0.78, 1.08)	1.00 (0.84, 1.17)	<0.001

Characteristic	Total (n =5,700)	< 0.31 (n =1,909)	0.31-0.47 (n =1,897)	> 0.47 (n =1,894)	*p*-value
**eGFR, ml/min/1.73m^2**	78.5 (65.0, 91.6)	81.2 (68.0, 93.6)	78.4 (65.3, 90.8)	76.1 (62.7, 89.6)	<0.001
**Lymphocytes, ×10^9/L**	1.70 (1.40, 2.10)	1.60 (1.30, 1.90)	1.70 (1.40, 2.10)	1.90 (1.50, 2.40)	<0.001
**Monocytes, ×10^9/L**	0.50 (0.40, 0.70)	0.40 (0.30, 0.50)	0.50 (0.50, 0.60)	0.70 (0.60, 0.80)	<0.001
**Neutrophils, ×10^9/L**	3.70 (2.90, 4.70)	3.10 (2.50, 3.90)	3.70 (3.10, 4.60)	4.50 (3.60, 5.50)	<0.001
**Platelets, ×10^9/L**	228 (191, 270)	225 (186, 264)	233 (194, 272)	226 (191, 272)	0.006
**LHR**	1.23 (0.89, 1.67)	0.88 (0.69, 1.13)	1.23 (0.97, 1.58)	1.72 (1.34, 2.18)	<0.001
**MHR**	0.38 (0.28, 0.53)	0.24 (0.20, 0.28)	0.39 (0.35, 0.42)	0.59 (0.53, 0.71)	<0.001
**NHR**	2.67 (1.86, 3.78)	1.75 (1.34, 2.33)	2.67 (2.10, 3.33)	3.96 (3.10, 5.14)	<0.001
**PHR**	162.4 (126.1, 206.8)	127.7 (100.5, 158.2)	165.2 (134.9, 200.0)	202.6 (165.0, 250.8)	<0.001
**Hypertension**	4,020 (67.5%)	1,277 (61.6%)	1,350 (67.5%)	1,393 (73.6%)	<0.001
**Diabetes**	1,957 (30.0%)	511 (21.2%)	644 (29.0%)	802 (40.0%)	<0.001
**CVD**	1,380 (23.7%)	306 (15.3%)	450 (22.5%)	624 (33.7%)	<0.001

Table S6. Baseline characteristics of the study population stratified by PHR tertiles.

**Characteristic**	**Total** (n =5,700)	**< 140.4** (n =1,900)	**140.4-192.7** (n =1,901)	**> 192.7** (n =1,899)	***p*-value**
**Age, years**	68 (64, 75)	70.0 (64.0, 77.0)	69.0 (63.0, 75.0)	67.0 (63.0, 74.0)	<0.001
**Sex**					<0.001
Male	2,853 (45.6%)	845.0 (39.8%)	979.0 (46.5%)	1,029.0 (50.9%)	
Female	2,847 (54.4%)	1,055.0 (60.2%)	922.0 (53.5%)	870.0 (49.1%)	
**Race**					0.003
Mexican American	781 (3.8%)	203.0 (3.2%)	272.0 (4.2%)	306.0 (4.3%)	
Black	998 (8.2%)	390.0 (9.2%)	314.0 (7.6%)	294.0 (7.6%)	
White	3,084 (79.5%)	1,047.0 (81.0%)	1,007.0 (78.4%)	1,030.0 (79.2%)	
Others	837 (8.4%)	260 (6.7%)	308 (9.8%)	269 (9.0%)	
**Education levels**					<0.001
Under high school	952 (8.8%)	277.0 (7.5%)	304.0 (8.5%)	371.0 (10.6%)	
High school or equivalent	2,195 (36.8%)	662.0 (33.2%)	752.0 (37.1%)	781.0 (40.4%)	
Above high school	2,553 (54.4%)	961.0 (59.3%)	845.0 (54.4%)	747.0 (49.0%)	
**Family income poverty ratio**					0.004
<1	917 (9.1%)	279.0 (8.1%)	276.0 (8.4%)	362.0 (11.0%)	
1-3	2,702 (41.2%)	880.0 (38.9%)	903.0 (41.1%)	919.0 (43.7%)	
≥3	2,081 (49.7%)	741.0 (53.0%)	722.0 (50.5%)	618.0 (45.3%)	
**Marital status**					0.4
Married or living with a partner	3,465 (65.1%)	1,096.0 (63.6%)	1,204.0 (66.2%)	1,165.0 (65.6%)	

Others	2,235 (34.9%)	804.0 (36.4%)	697.0 (33.8%)	734.0 (34.4%)	
**Smoking status**					<0.001
Never smoker	2,740 (47.8%)	999.0 (51.6%)	916.0 (47.9%)	825.0 (43.6%)	
Former smoker	2,276 (41.1%)	731.0 (41.5%)	788.0 (42.9%)	757.0 (39.0%)	
Current smoker	684 (11.1%)	170.0 (7.0%)	197.0 (9.2%)	317.0 (17.5%)	
**Drinking status**					<0.001
Nondrinker	2,719 (41.6%)	830.0 (37.1%)	888.0 (40.5%)	1,001.0 (47.7%)	
Low-to-moderate drinker	2,113 (42.4%)	742.0 (44.3%)	725.0 (43.2%)	646.0 (39.4%)	
Heavy drinker	868 (16.0%)	328.0 (18.6%)	288.0 (16.3%)	252.0 (12.9%)	
**BMI, kg/m^2**	27.9 (24.7, 32.1)	26.6 (23.6, 30.2)	28.2 (25.1, 32.2)	29.6 (26.0, 33.9)	<0.001
**BMI category**					<0.001
Normal weight (<25)	1,532 (26.8%)	691.0 (36.6%)	459.0 (24.1%)	382.0 (18.9%)	
Overweight (25-30)	2,099 (36.2%)	681.0 (37.3%)	726.0 (37.6%)	692.0 (33.5%)	
Obesity (≥30)	2,069 (37.0%)	528.0 (26.1%)	716.0 (38.3%)	825.0 (47.7%)	
**TC, mmol/L**	4.97 (4.27, 5.74)	5.15 (4.42, 5.87)	4.97 (4.22, 5.64)	4.78 (4.14, 5.61)	<0.001
**TG, mmol/L**	1.26 (0.90, 1.78)	0.96 (0.72, 1.30)	1.30 (0.98, 1.75)	1.68 (1.21, 2.22)	<0.001
**LDL, mmol/L**	2.87 (2.25, 3.49)	2.79 (2.20, 3.44)	2.90 (2.28, 3.49)	2.87 (2.20, 3.54)	0.12
**HDL, mmol/L**	1.40 (1.14, 1.73)	1.78 (1.53, 2.09)	1.37 (1.19, 1.58)	1.14 (0.98, 1.29)	<0.001
**HbA1c, %**	5.70 (5.40, 6.10)	5.60 (5.40, 5.90)	5.70 (5.40, 6.10)	5.80 (5.50, 6.20)	<0.001
**Fast glucose, mmol/L**	5.83 (5.38, 6.55)	5.72 (5.24, 6.22)	5.88 (5.40, 6.61)	6.05 (5.50, 6.88)	<0.001
**Serum creatinine, mg/dl**	0.91 (0.78, 1.09)	0.90 (0.76, 1.07)	0.90 (0.79, 1.10)	0.94 (0.79, 1.10)	0.002
**eGFR, ml/min/1.73m^2**	78.5 (65.0, 91.6)	78.4 (65.3, 91.2)	79.0 (65.0, 91.4)	78.0 (64.5, 92.5)	>0.9
**Lymphocytes, ×10^9/L**	1.70 (1.40, 2.10)	1.60 (1.30, 2.00)	1.80 (1.40, 2.10)	1.90 (1.50, 2.40)	<0.001

**Monocytes, ×10^9/L**	0.50 (0.40, 0.70)	0.50 (0.40, 0.60)	0.50 (0.40, 0.60)	0.60 (0.50, 0.70)	<0.001
**Neutrophils, ×10^9/L**	3.70 (2.90, 4.70)	3.30 (2.60, 4.10)	3.70 (3.00, 4.70)	4.30 (3.40, 5.40)	<0.001
**Platelets, ×10^9/L**	228 (191, 270)	192 (165, 225)	227 (198, 259)	273 (237, 314)	<0.001
**LHR**	1.23 (0.89, 1.67)	0.88 (0.69, 1.13)	1.25 (1.01, 1.60)	1.68 (1.29, 2.17)	<0.001
**MHR**	0.38 (0.28, 0.53)	0.28 (0.22, 0.37)	0.39 (0.30, 0.50)	0.53 (0.39, 0.67)	<0.001
**NHR**	2.67 (1.86, 3.78)	1.85 (1.38, 2.52)	2.69 (2.05, 3.50)	3.78 (2.86, 5.00)	<0.001
**PHR**	162.4 (126.1, 206.8)	112.7 (94.4, 128.6)	164.9 (152.9, 176.6)	230.8 (209.3, 268.5)	<0.001
**Hypertension**	4,020 (67.5%)	1,300.0 (62.8%)	1,338.0 (67.9%)	1,382.0 (72.2%)	<0.001
**Diabetes**	1,957 (30.0%)	538.0 (22.3%)	679.0 (32.2%)	740.0 (36.1%)	<0.001
**CVD**	1,380 (23.7%)	538.0 (22.3%)	679.0 (32.2%)	740.0 (36.1%)	0.056
